# Fruit, Vegetable, and Sugar-Sweetened Beverage Intake Among Young Children, by State — United States, 2021

**DOI:** 10.15585/mmwr.mm7207a1

**Published:** 2023-02-17

**Authors:** Heather C. Hamner, Carrie A. Dooyema, Heidi M. Blanck, Rafael Flores-Ayala, Jessica R. Jones, Reem M. Ghandour, Ruth Petersen

**Affiliations:** ^1^Division of Nutrition, Physical Activity, and Obesity, National Center for Chronic Disease Prevention and Health Promotion, CDC; ^2^Office of Epidemiology and Research, Maternal and Child Health Bureau, Health Resources and Services Administration, Rockville, Maryland.

Good nutrition in early childhood supports optimal growth, development, and health (*1*). Federal guidelines support a dietary pattern with daily fruit and vegetable consumption and limited added sugars, including limited consumption of sugar-sweetened beverages (*1*). Government-published dietary intake estimates for young children are outdated at the national level and unavailable at the state level. CDC analyzed data from the 2021 National Survey of Children’s Health (NSCH)* to describe how frequently, according to parent report, children aged 1–5 years (18,386) consumed fruits, vegetables, and sugar-sweetened beverages, nationally and by state. During the preceding week, approximately one in three (32.1%) children did not eat a daily fruit, nearly one half (49.1%) did not eat a daily vegetable, and more than one half (57.1%) drank a sugar-sweetened beverage at least once. Estimates of consumption varied by state. In 20 states, more than one half of children did not eat a vegetable daily during the preceding week. In Vermont, 30.4% of children did not eat a daily vegetable during the preceding week, compared with 64.3% in Louisiana. In 40 states and the District of Columbia, more than one half of children drank a sugar-sweetened beverage at least once during the preceding week. The percentage of children drinking sugar-sweetened beverages at least once during the preceding week ranged from 38.6% in Maine to 79.3% in Mississippi. Many young children are not consuming fruits and vegetables daily and are regularly consuming sugar-sweetened beverages. Federal nutrition programs and state policies and programs can support improvements in diet quality by increasing access to and availability of fruits and vegetables and healthy beverages in places where young children live, learn, and play.

NSCH uses paper- and web-based questionnaires to collect information on the health and well-being of U.S. children and adolescents aged <18 years; it is funded and directed by the Health Resources and Services Administration’s Maternal Child Health Bureau and conducted by the U.S. Census Bureau. Households are randomly sampled from the Census Bureau’s Master Address File and contacted via mail to identify those with at least one child or adolescent aged <18 years. One child or adolescent per household is selected, and an age-specific questionnaire is completed by a household adult familiar with the selected child or adolescent’s health and health care. Children aged <6 years are oversampled. The surveys were available in English and Spanish. The 2021 weighted overall response and interview completion rates^†^ were 40.3% and 79.5%, respectively. Data were collected during June 2021–January 2022.

Respondents were asked three questions about children aged 1–5 years regarding the frequency of consuming fruits,^§^ vegetables,^¶^ and sugar-sweetened beverages** during the preceding week. Response options included the following: did not consume item, 1–3 times in the preceding week, 4–6 times in the preceding week, 1 time per day, 2 times per day, and ≥3 times per day. Categories were recoded to provide an estimate of daily (≥1 time per day in preceding week) or less than daily (<1 time per day in preceding week) consumption of fruit and vegetables. Categories of sugar-sweetened beverages were dichotomized to indicate consumption at least once or no consumption during the preceding week. Among the 18,830 children aged 1–5 years, 444 (2.4%) were missing data on at least one item and were excluded, leaving a final analytic sample of 18,386. Weighted percentages are presented overall, by child’s age, race and ethnicity, household food sufficiency,^††^ and by state, using SPSS Complex Samples (version 1.0.0.1401; IBM) to account for the sampling procedures. Pearson Chi-square tests of independence were used to identify differences within each outcome by sociodemographic characteristics. This activity was reviewed by CDC and was conducted consistent with applicable federal law and CDC policy.^§§^

In 2021, 32.1% of children aged 1–5 years did not eat a daily fruit, and 49.1% did not eat a daily vegetable during the preceding week; 57.1% drank a sugar-sweetened beverage at least once during the preceding week (Table 1). Daily consumption of fruit and vegetables and weekly consumption of sugar-sweetened beverages differed by age, race and ethnicity, and household food sufficiency. Children aged 1 year were more likely than were older children to eat either a daily fruit or a daily vegetable during the preceding week and were less likely to drink a sugar-sweetened beverage (chi-square p<0.05). The percentage of children who did not eat a daily fruit or vegetable was highest among non-Hispanic Black (Black) children and lowest among non-Hispanic White (White) children. Drinking a sugar-sweetened beverage at least once during the preceding week ranged from 47.5% among multiracial non-Hispanic children to 71.7% among Black children. Compared with children living in food-sufficient households, those living in households with marginal or low food sufficiency were less likely to eat either a daily fruit or vegetable and were more likely to consume sugar-sweetened beverages during the preceding week.

**TABLE 1:** Percentage of children aged 1–5 years who consumed fruit, vegetables, or sugar-sweetened beverages during the preceding week, by sociodemographic characteristics — National Survey of Children's Health, United States, 2021

Characteristic	Total no. (unweighted)*	% (95% CI)^†^		
		Fruit	Vegetables	Sugar-sweetened beverages
		Less than daily		At least once weekly
**United States**	**18,386**	**32.1 (30.4–33.7)**	**49.1 (47.3–50.8)**	**57.1 (55.4–58.8)**
**Child age, yrs**				
1	**2,438**	25.4 (21.5–29.7)^§^	43.9 (39.4–48.4)^§^	30.9 (26.7–35.5)^§^
2	**4,225**	31.6 (28.3–35.1)	47.7 (44.3–51.2)	51.4 (48.0–54.9)
3	**3,799**	31.8 (28.3–35.5)	49.7 (45.8–53.6)	61.4 (57.7–65.0)
4	**3,974**	34.9 (31.1–39.0)	50.5 (46.5–54.6)	67.8 (64.2–71.2)
5	**3,950**	36.2 (32.7–39.9)	53.2 (49.4–57.0)	72.3 (68.9–75.5)
**Race and ethnicity^¶^**				
Asian, non-Hispanic	**1,046**	42.2 (34.9–49.8)	47.5 (40.3–54.7)	56.2 (49.1–63.0)
Black or African American, non-Hispanic	**1,061**	50.7 (45.1–56.4)	64.8 (59.2–70.0)	71.7 (66.0–76.8)
Hispanic or Latino	**2,407**	33.6 (29.3–38.2)^§^	53.7 (48.9–58.5)^§^	67.2 (62.8–71.3)^§^
White, non-Hispanic	**12,305**	26.1 (24.6–27.7)	43.4 (41.6–45.2)	49.6 (47.8–51.5)
Multiracial, non-Hispanic	**1,567**	26.8 (22.6–31.6)	44.1 (39.0–49.3)	47.5 (42.5–52.6)
Food situation in the past 12 months**				
Food sufficiency: could always afford to eat good nutritious meals	**14,483**	29.6 (27.7–31.5)^§^	46.5 (44.5–48.5)^§^	53.1 (51.1–55.1)^§^
Marginal food sufficiency: could always afford enough to eat but not always the kinds of foods we should eat	**3,215**	36.9 (33.3–40.8)	56.2 (51.9–60.4)	69.2 (65.3–72.8)
Low food sufficiency: sometimes or often could not afford enough to eat	**348**	46.4 (36.4–56.6)^††^	59.0 (48.3–68.9)^††^	70.9 (61.2–79.0)

* Denominators might not sum to total because of missing sociodemographic data.

^†^ Percentages are weighted to account for complex survey design and adjusted for the probability of selection, nonresponse, and demographic factors to represent noninstitutionalized children in the United States and in each jurisdiction.

^§^ For each outcome, a Pearson’s chi-square test of independence was done to identify differences by sociodemographic characteristics; p<0.05 was considered statistically significant.

^¶^ Persons who indicated they were American Indian or Alaska Native, or Native Hawaiian or other Pacific Islander were included in the Multiracial, non-Hispanic group because point estimates for all three dietary outcomes were unstable and needed to be interpreted with caution.

** Food sufficiency was assessed by asking, “Which of the following best describes your household’s ability to afford the food you need during the past 12 months?” Response options were recoded to Food sufficiency (could always afford to eat good nutrition meals), Marginal food sufficiency (could always afford enough to eat but not always the kinds of foods we should eat), or Low food sufficiency (sometimes or often we could not afford enough to eat).

^††^ Based on National Survey of Children's Health data, presentation criteria states that if the 95% CI width is >20 percentage points or 1.2 times the estimate (approximate relative SE >30%), data should be flagged for poor reliability and/or present a measure of statistical reliability (e.g., CI or statistical significance testing) to promote appropriate interpretation. https://www.census.gov/programs-surveys/nsch/data/datasets.html

Estimates of intake varied by state (Table 2). The percentage of children who did not eat fruit daily during the preceding week ranged from 16.3% in Vermont to 49.9% in Louisiana. Vegetable intake also varied: 30.4% of children in Vermont did not eat a daily vegetable, compared with 64.3% in Louisiana. The percentage of children who consumed a sugar-sweetened beverage at least once during the preceding week ranged from 38.8% (Maine) to 79.3% (Mississippi). In 20 states, more than one half of children did not eat a daily vegetable during the preceding week (Figure). In 40 states and the District of Columbia, more than one half of children drank a sugar-sweetened beverage at least once during the preceding week.

**TABLE 2:** Percentage of children aged 1–5 years who consumed fruit, vegetables, or sugar-sweetened beverages during the preceding week, by state — National Survey of Children's Health, 2021

Jurisdiction	Total no. (unweighted)	% (95% CI)*		
		Fruit	Vegetables	Sugar-sweetened beverages
		Less than daily		At least once weekly
Alabama	**339**	39.3 (32.0–47.2)	57.3 (49.6–64.8)	66.5 (59.7–72.7)
Alaska	**350**	21.6 (15.5–29.3)	50.0 (41.8–58.1)	54.9 (46.7–62.7)
Arizona	**315**	30.0 (22.2–39.2)	50.6 (41.5–59.7)	59.6 (50.4–68.1)
Arkansas	**327**	36.3 (28.5, 44.8)	51.5 (42.9–60.1)	66.1 (57.8–73.6)
California	**342**	32.3 (24.9–40.7)	50.5 (42.5–58.4)	53.9 (46.0–61.5)
Colorado	**503**	25.6 (20.3–31.8)	47.2 (40.7–53.8)	56.1 (49.7–62.2)
Connecticut	**368**	33.5 (25.2–42.9)	48.5 (40.1–56.9)	42.5 (34.2–51.3)
Delaware	**340**	28.7 (22.6–35.6)	53.6 (45.5–61.5)	54.2 (46.1–62.0)
District of Columbia	**388**	37.0 (27.5–47.6)^†^	43.9 (34.3–53.9)	51.1 (41.5–60.7)
Florida	**329**	34.4 (26.7–43.1)	52.0 (43.4–60.5)	57.5 (48.9–65.6)
Georgia	**453**	37.6 (31.1–44.7)	47.9 (41.2–54.7)	62.5 (55.9–68.6)
Hawaii	**400**	38.6 (31.7–46.1)	55.5 (48.0–62.6)	54.3 (46.9–61.5)
Idaho	**294**	38.3 (29.9–47.4)	50.4 (42.0–58.8)	68.6 (60.4–75.9)
Illinois	**379**	31.0 (23.8–39.1)	49.8 (41.6–58.0)	52.3 (44.3–60.2)
Indiana	**354**	43.1 (35.6–51.0)	53.2 (45.5–60.8)	66.7 (59.5–73.1)
Iowa	**358**	32.3 (25.5–40.0)	49.9 (42.4–57.4)	54.7 (46.9, 62.2)
Kansas	**372**	34.8 (27.5–43.0)	43.4 (36.1–51.1)	56.2 (48.6–63.6)
Kentucky	**337**	42.7 (34.8–51.0)	54.4 (46.2–62.4)	59.0 (50.6–66.9)
Louisiana	**330**	49.9 (41.9–57.9)	64.3 (56.3–71.6)	70.2 (62.2–77.1)
Maine	**394**	20.0 (14.8–26.3)	33.9 (27.7–40.8)	38.6 (32.5–45.1)
Maryland	**281**	26.3 (19.7–34.3)	46.4 (37.6, 55.5)	57.6 (48.7–66.1)
Massachusetts	**311**	20.1 (14.8–26.8)	46.7 (38.3–55.3)	46.7 (38.7–54.8)
Michigan	**324**	31.0 (24.4–38.5)	44.3 (37.1–51.7)	53.2 (45.6–60.8)
Minnesota	**304**	22.1 (15.4–30.6)	41.2 (33.0–49.9)	55.5 (47.1–63.5)
Mississippi	**323**	47.3 (38.7–56.0)	55.8 (46.8–64.4)	79.3 (72.1–85.0)
Missouri	**336**	37.0 (29.4–45.2)	44.3 (36.4–52.5)	60.1 (52.0–67.7)
Montana	**353**	29.2 (21.9–37.7)	42.8 (35.1–50.8)	59.0 (50.3–67.2)
Nebraska	**397**	33.5 (26.4–41.6)	52.2 (44.4–59.8)	59.5 (51.9–66.5)
Nevada	**314**	33.4 (25.9–41.8)	44.2 (35.8–52.8)	57.0 (48.2–65.4)
New Hampshire	**313**	22.2 (16.3–29.6)	38.5 (31.3–46.2)	41.7 (34.2–49.6)
New Jersey	**356**	32.6 (25.3–40.7)	57.1 (49.2–64.6)	53.2 (45.2–60.9)
New Mexico	**305**	41.1 (31.7–51.2)	47.7 (38.0–57.6)	66.2 (56.2–75.0)
New York	**315**	37.8 (30.4–45.9)	55.6 (47.6–63.3)	49.3 (41.7–57.0)
North Carolina	**304**	26.4 (19.8–34.2)	49.1 (40.2–58.1)	54.0 (45.0–62.7)
North Dakota	**325**	32.7 (25.9–40.4)	44.3 (37.5–51.5)	63.6 (56.3–70.4)
Ohio	**435**	27.1 (21.8–33.2)	45.8 (39.5–52.3)	51.1 (45.1–57.1)
Oklahoma	**342**	37.5 (29.6–46.0)	57.5 (49.0–65.5)	72.6 (64.9–79.1)
Oregon	**908**	26.1 (21.9–30.7)	43.2 (38.4–48.2)	48.6 (43.7–53.5)
Pennsylvania	**320**	27.4 (20.2–36.0)	44.5 (36.1–53.1)	44.9 (36.4–53.7)
Rhode Island	**346**	34.1 (26.1–43.0)	56.2 (47.8–64.3)	52.6 (44.1–60.9)
South Carolina	**321**	34.3 (27.1–42.2)	47.5 (39.0–56.1)	61.5 (53.1–69.3)
South Dakota	**353**	36.7 (30.0–43.9)	52.7 (45.6–59.7)	58.1 (51.0–65.0)
Tennessee	**336**	36.2 (28.3–44.9)	42.5 (34.4–50.9)	72.1 (64.5–78.6)
Texas	**315**	29.9 (23.3–37.4)	47.3 (38.9–55.9)	68.8 (60.8–75.8)
Utah	**377**	28.4 (23.3–34.2)	52.5 (46.1–58.8)	66.6 (60.3–72.3)
Vermont	**329**	16.3 (11.8–22.1)	30.4 (23.6–38.3)	41.3 (33.8–49.2)
Virginia	**302**	30.8 (23.1–39.7)	51.8 (43.0–60.5)	45.7 (36.9–54.7)
Washington	**359**	19.4 (14.0–26.3)	35.5 (28.6–42.9)	46.3 (38.8–53.9)
West Virginia	**342**	40.0 (32.4–48.2)	49.6 (41.6–57.7)	64.9 (57.2–71.9)
Wisconsin	**603**	26.3 (21.6–31.6)	44.9 (39.4–50.6)	50.9 (45.3–56.4)
Wyoming	**265**	25.5 (19.7–32.4)	45.4 (37.2–53.8)	64.3 (55.4–72.4)

* Percentages are weighted to account for complex survey design and adjusted for the probability of selection, nonresponse, and demographic factors to represent noninstitutionalized children in the United States and in each jurisdiction.

^†^ Based on National Survey of Children's Health data, presentation criteria states that if the 95% CI width exceeds 20 percentage points or 1.2 times the estimate (approximate relative SE >30%), data should be flagged for poor reliability and/or present a measure of statistical reliability (e.g., CI or statistical significance testing) to promote appropriate interpretation. https://www.census.gov/programs-surveys/nsch/data/datasets.html

**FIGURE:**
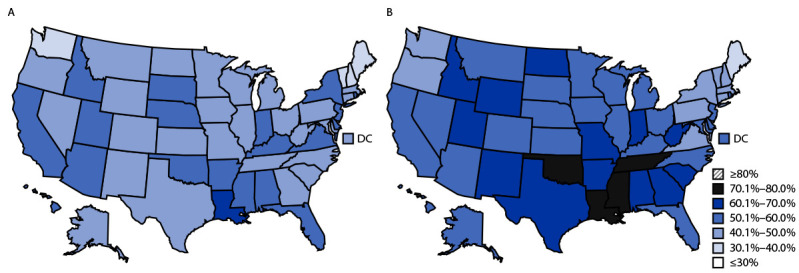
Percentage of children aged 1–5 years who (A) ate vegetables* less than once a day during the preceding week or (B) drank at least one sugar-sweetened beverage† in the preceding week, by state — United States, 2021 **Abbreviation:** DC = District of Columbia. * Percentage of children aged 1–5 years who ate vegetables less than once a day during the preceding week: ≤30%, n = 0; 30.1%–40.0%, n = 4; 40.1%–50.0%, n = 27; 50.1%–60.0%, n = 19; 60.1%–70.0%, n = 1; 70.1%–80.0%, n = 0; and ≥80%, n = 0. ^†^ Percentage of children aged 1–5 years who drank at least one sugar-sweetened beverage in the preceding week: ≤30%, n = 0; 30.1%–40.0%, n = 1; 40.1%–50.0%, n = 9; 50.1%–60.0%, n = 24; 60.1%–70.0%, n = 13; 70.1%–80.0%, n = 4; and ≥80%, n = 0.

## Discussion

In 2021, nearly one third (32.1%) of children aged 1–5 years did not eat a daily fruit, and nearly one half (49.1%) did not eat a daily vegetable during the preceding week; more than one half (57.1%) drank a sugar-sweetened beverage at least once during the preceding week. The percentage of children who did not eat a daily fruit or vegetable was higher among those who were aged 2–5 years, Black, or lived in households with limited food sufficiency. Similar patterns were seen for consumption of sugar-sweetened beverages. State-level estimates for all three dietary practices varied widely.

Young children need specific nutrients to support their optimal growth and development (*1*,*2*). A diet rich in fruits and vegetables can help provide these nutrients (*1*). Limiting or reducing foods and beverages higher in added sugars, including sugar-sweetened beverages, is important because added sugars are associated with increased risk of obesity, dental caries, diabetes, and cardiovascular disease (*3*–*6*). These data provide current assessments that states can use to prioritize actions to improve early childhood nutrition.

Programs and policies can support efforts to improve fruit and vegetable intake and reduce consumption of sugar-sweetened beverages among young children. The U.S. Department of Agriculture’s (USDA) Special Supplemental Nutrition Program for Women, Infants, and Children (WIC), a program for low-income families, provides nutrition education, supplemental foods, including fruits and vegetables, and referrals to health care services.^¶¶^ WIC is an important conduit for reaching participating families with nutrition education messages and healthy supplemental foods. Nutrition standards in early care and education (ECE) systems and in the charitable food system can support access to fruits and vegetables and help limit the intake of foods and beverages with added sugars. CDC supports*** system-level efforts, including standards in the ECE state licensing regulations that support healthy eating, professional development opportunities for ECE staff members, and programs that provide young children an opportunity to learn about food, agriculture, and gardening through hands-on experiences. Federally funded programs, such as produce voucher programs and the Child and Adult Care Food Program, have resulted in serving more nutritious foods to children (*7*). Federal nutrition programs are a system-level approach that can improve diet quality for young children. The effectiveness of federal, state, or local-level programs could be enhanced by education emphasizing the importance of daily fruit and vegetable consumption and reducing sugar-sweetened beverage intake across multiple settings. Examples of existing programs that support such educational efforts include home visiting programs,^†††^ Healthy Start,^§§§^ and USDA’s Supplemental Nutrition Assistance Program Education.^¶¶¶^ Health care providers can also convey the importance of healthy dietary choices through anticipatory guidance (i.e., Bright Futures****) and regular screening and counseling on food and nutrition security and key dietary behaviors during health care encounters. Understanding how access, affordability, and taste preferences influence diet for young children (*8*,*9*) could help tailor programmatic, communication, and education efforts.

The findings in this report are subject to at least four limitations. First, children’s dietary intake was reported by an adult who might not know everything a child ate. Second, frequency of intake was assessed, not the amount consumed; therefore, intake cannot be tied to a dietary recommendation. Third, information collection occurred in English or Spanish and might not represent families who speak other languages. Finally, questions reflect intake during the preceding week and might not represent usual intake.

With renewed national focus on nutrition, hunger, and health and the call to improve food and nutrition security,^††††^ these data provide information for decision makers and practitioners to ensure that young children have an opportunity for their healthiest start. Collectively, programs and policies aimed at supporting nutrition for young children could lead to improvements in dietary quality and support optimal growth and health.

**Summary**

**What is already known about this topic?**

Good nutrition is important for young children’s health. Dietary guidelines support daily intake of fruits and vegetables and limited intake of sugar-sweetened beverages.

**What is added by this report?**

Many children aged 1–5 years, are not eating fruits and vegetables daily and are regularly drinking sugar-sweetened beverages. In 20 states, more than one half of children did not eat a vegetable daily during the preceding week. In 40 states and the District of Columbia, more than one half of children drank a sugar-sweetened beverage at least once during the preceding week.

**What are the implications for public health practice?**

Emphasizing the importance of healthy dietary practices in existing programs and policies that affect young children could improve their nutrition and support optimal growth and health.

## Footnotes

*
https://mchb.hrsa.gov/data-research/national-survey-childrens-health

† The weighted overall response rate is the probability that an address progresses through the three major stages of survey completion: resolution, screener, and topical questionnaire. The weighted interview completion rate is the probability that a household that initiates the survey will complete it.

§ Fruit includes fresh, frozen, or canned. It does not include juice.

¶ Vegetables includes fresh, frozen, or canned. It does not include french fries, fried potatoes, or potato chips.

** Sugar-sweetened beverages includes soda, fruit drinks, sports drinks, or sweet tea. It does not include 100% fruit juice.

†† Food sufficiency was assessed by asking, “Which of the following best describes your household’s ability to afford the food you need during the past 12 months?” Response options were recoded to food sufficiency (could always afford to eat good nutrition meals) marginal food sufficiency (could always afford enough to eat but not always the kinds of foods we should eat), and low food sufficiency (sometimes or often we could not afford enough to eat).

§§ 45 C.F.R. part 46, 21 C.F.R. part 56; 42 U.S.C. Sect. 241(d); 5 U.S.C. Sect.552a; 44 U.S.C. Sect. 3501 et seq.

¶¶
https://www.fns.usda.gov/wic

***
https://www.cdc.gov/obesity/strategies/early-care-education/cdc-funded-ece-projects.html

†††
https://mchb.hrsa.gov/programs-impact/programs/home-visiting/maternal-infant-early-childhood-home-visiting-miechv-program

§§§
https://mchb.hrsa.gov/programs-impact/healthy-start

¶¶¶
https://www.fns.usda.gov/snap/snap-ed

****
https://www.aap.org/en/practice-management/bright-futures/bright-futures-materials-and-tools/bright-futures-guidelines-and-pocket-guide/

††††
https://www.whitehouse.gov/wp-content/uploads/2022/09/White-House-National-Strategy-on-Hunger-Nutrition-and-Health-FINAL.pdf
